# Using WhatsApp for Nutrition Surveillance Among Children Under 5 Years in West Java, Indonesia: Cross-Sectional Survey and Feasibility Study

**DOI:** 10.2196/58752

**Published:** 2025-05-15

**Authors:** Dewi Nur Aisyah, Chyntia Aryanti Mayadewi, Astri Utami, Fauziah Mauly Rahman, Nathasya Humaira Adriani, Erlangga Al Farozi, Meldi Hafizh Sayoko, Aulia Chairunisa, Liza Restiana, Logan Manikam, Zisis Kozlakidis

**Affiliations:** 1 Department of Epidemiology and Public Health, Institute of Epidemiology and Health Care, University College London, 1-19 Torrington Place, London, WC1E 7HB, United Kingdom, 44 (0)207 679 2000; 2 Digital Transformation Office, Ministry of Health, Republic of Indonesia, Jakarta, Indonesia; 3 Aceso Global Health Consultants Pte Limited, #32-02, International Plaza, Singapore, 079903, Singapore; 4 Department of Public Health, Monash University Indonesia, Tangerang, Indonesia; 5 School of Computer Science, Faculty of Engineering, University of Sydney, Camperdown, NSW, Australia; 6 Department of Science, Technology and Innovation Studies, School of Social and Political Science, University of Edinburgh, Edinburgh, United Kingdom; 7 Ciderum Community Health Center, Caringin Subdistrict, Bogor, Indonesia; 8 International Agency for Research on Cancer World Health Organization, Lyon, France

**Keywords:** WhatsApp, Posyandu, nutrition surveillance, nutrition, nutritional, mHealth, mobile health, app, anthropometric, data collection, surveillance, chatbots, digital, digital health, digital technology, digital intervention, pediatric, infant, baby, neonatal, toddler, child

## Abstract

**Background:**

Large-scale programs involving nutrition-specific interventions have been carried out in Indonesia as a community-based approach at the primary care level across cities and districts, throughout the age-specific target population (ie, children under 5 years).

**Objective:**

The aim of this paper is to describe the potential use of WhatsApp as a tool for recording and monitoring the growth of children under 5 years by Posyandu (Pos Pelayanan Terpadu or community-based health service post), investigating its potential in enhancing health programs and services.

**Methods:**

Data were collected from Posyandu cadres in Bogor District, West Java, from March to June 2022. The anthropometric measurement data were reported in real time through a WhatsApp chatbot, automatically analyzed by the system, and presented in a structured dashboard. A qualitative assessment was carried out using a cross-sectional survey conducted from March to July 2022.

**Results:**

The study involved 42 Posyandu in 3 villages, engaging 282 staff, and the WhatsApp chatbot recorded anthropometric data for 4571 children under 5 years. The qualitative assessment indicated widespread system utilization, with 50% (45/90) affirming comprehensive data input. Additionally, 66.4% (83/129) found the system easy to use, and 66.7% (82/123) expressed clarity in comprehending variables. Moreover, 75.6% (93/123) found the data input flow easily understood, and 74% (91/123) suggested that the system contributed to enhancing Posyandu activities and the quality of data reporting. Regarding staff proficiency, 63.5% (80/126) affirmed their adeptness in using the system, and 71% (88/124) asserted their high capability in providing training to colleagues.

**Conclusions:**

The potential use of WhatsApp as a surveillance tool for recording children’s nutritional status is promising, suggesting broader applications within health programs. Nonetheless, this expansion requires additional improvements, including human resource preparation, Posyandu infrastructure development, and strong regulatory support.

## Introduction

Stunting is a condition described as a linear growth failure in which children are too short for their age, and it has been deemed a significant public health issue globally [1-3]. Children with stunted growth are at heightened risk of irreversible loss of growth, suboptimal cognitive development, and elevated risk of morbidities and mortalities in adulthood [3,4]. Suboptimal cognitive development leads to lower cognitive skills, resulting in poor academic performance and lower incomes [4,5]. Although there was a significant decrease in prevalence within the last decade, 148.1 million (22.3%) children under 5 years (CU5) were estimated as having stunted growth in 2022 [1]. According to the Indonesian Nutritional Status Survey (Survei Status Gizi Indonesia) in 2023, the prevalence of children with stunted growth in Indonesia in 2022 was 21.6%, thus similar to the global burden [1,6,7].

To accurately describe a population’s nutritional status, particularly the at-risk groups, establishing a nutritional surveillance system becomes pivotal [8]. Such a nutritional surveillance system needs to collect data on nutrition risk factors in a regular and timely manner from various sources, such as growth monitoring in health facilities, nutrition surveys for the population, as well as community-based sentinel monitoring [8-10]. In Indonesia, community-based sentinel sites for growth monitoring for CU5 are conducted through Posyandu (Pos Pelayanan Terpadu; community-based health service post), where the anthropometry measurements are done routinely to help obtain cohort nutritional status information of the children [11,12]. Posyandu serves as a community-based public health outreach initiative, managed and organized in partnership with the community, linking the latter to public health centers (Pusat Kesehatan Masyarakat [Puskesmas]), with the goal of improving access to basic health services, particularly for reducing maternal and child mortality rates [13]. Posyandu itself is integrated into the village social institutions, contributing to enhanced health services for the community [14].

Posyandu undertakes a comprehensive range of health services, consisting of but not limited to maternal and child health care, including immunization services; adolescent reproductive health services; older adult health services; disease control; environmental sanitation; promotion of healthy lifestyles; and diversification of food consumption [11]. These activities are carried out by dedicated and trained community health workers, also referred to as cadres, who are community members voluntarily devoting their time and efforts to organizing Posyandu activities [11]. Indonesia currently possesses a substantial network of more than 213,000 Posyandu, with an estimated 1,039,684 active volunteers nationwide [15]. Trained community health workers play a crucial role in the success of Posyandu and the reduction of malnutrition [16]. In 2021, a presidential decree by the Indonesian government focused on the acceleration of stunting reduction and emphasized the importance of growth and development monitoring services at Posyandu [17].

In general, the reporting process in Posyandu begins with the cadre registering the children and mother in the Posyandu’s registration book. It is then followed by the measurement of the height and body weight of the children, which are recorded in the Maternal and Child Health book as well as the Posyandu book [11,12]. After the child and the mother are given health education and other health services as needed, the data are input into an Excel file (Microsoft Corp). The file is then sent to the Puskesmas (public health center), where the workers begin inputting the data into the nutrition surveillance system. This process creates a delay in generating the nutritional status of the children and presents challenges such as difficulty for the cadres to plot the growth chart, possibility of loss of records, and potential inclusion of errors.

Mobile phones and internet usage are increasingly used for health programs, service delivery, and public health messaging [18]. Young adults are very likely to use mobile apps and the internet for health purposes [19]. Furthermore, in low- and middle-income countries (LMICs), mobile phone technologies are promising tools for enhancing primary health care services [20]. In 2021, 125.6% of the total population in Indonesia possessed a mobile phone (ie, there is more than 1 phone per person) [21], while in 2022, 66% of the population had used the internet [22], with the majority of apps used falling within the social and communication spectrum [23]. As of 2023, WhatsApp reached 120.35 million users in Indonesia with an average use time of 29 hours per month, making it the most used app nationally [24]. Aside from being a communication tool, WhatsApp is used for other purposes such as facilitating education (as a supporting tool for e-learning), as well as for health purposes such as for diagnostic and telemedicine support [25-31], though WhatsApp’s use as a survey tool remains limited.

Indonesia is considered a digital force in Southeast Asia [32], with highly advanced digital health services integration. With the launch of the Digital Health Transformation Blueprint, the government committed to enhancing public health through the means of digitization [33]. One of these digitization programs involves improving the quality and reach of surveillance of the nutritional status of CU5 conducted by community health workers. To this end, the Digital Transformation Office – Pusat Data dan Informasi (DTO-Pusdatin) team at the Ministry of Health carried out a pilot project using WhatsApp as a data input tool to monitor the growth status of CU5 during Posyandu activities. This paper investigates the potential use of WhatsApp as a tool for recording and monitoring the growth of CU5 in Posyandu and evaluates its application within the national framework of providing an improved national surveillance service.

## Methods

### Using WhatsApp for CU5 Nutrition Surveillance

#### Overview

There are varying capacities among Posyandu’s cadres in Indonesia with respect to technology; not all are acquainted with or adept at using mobile apps or information systems for nutrition surveillance recording and reporting. To address this, a novel approach is required to simplify the process of recording and reporting nutrition surveillance in Posyandu, especially that which is to be done by the cadres. In response to this need, the DTO-Pusdatin Ministry of Health introduced an innovation by creating a WhatsApp chatbot specifically designed for inputting CU5 measurement results by the cadres at Posyandu. A feasibility study to test the reporting system through the WhatsApp chatbot was conducted from March to June 2022 at the Ciderum Puskesmas, located in Bogor District, West Java Province, Indonesia.

**Figure 1.**
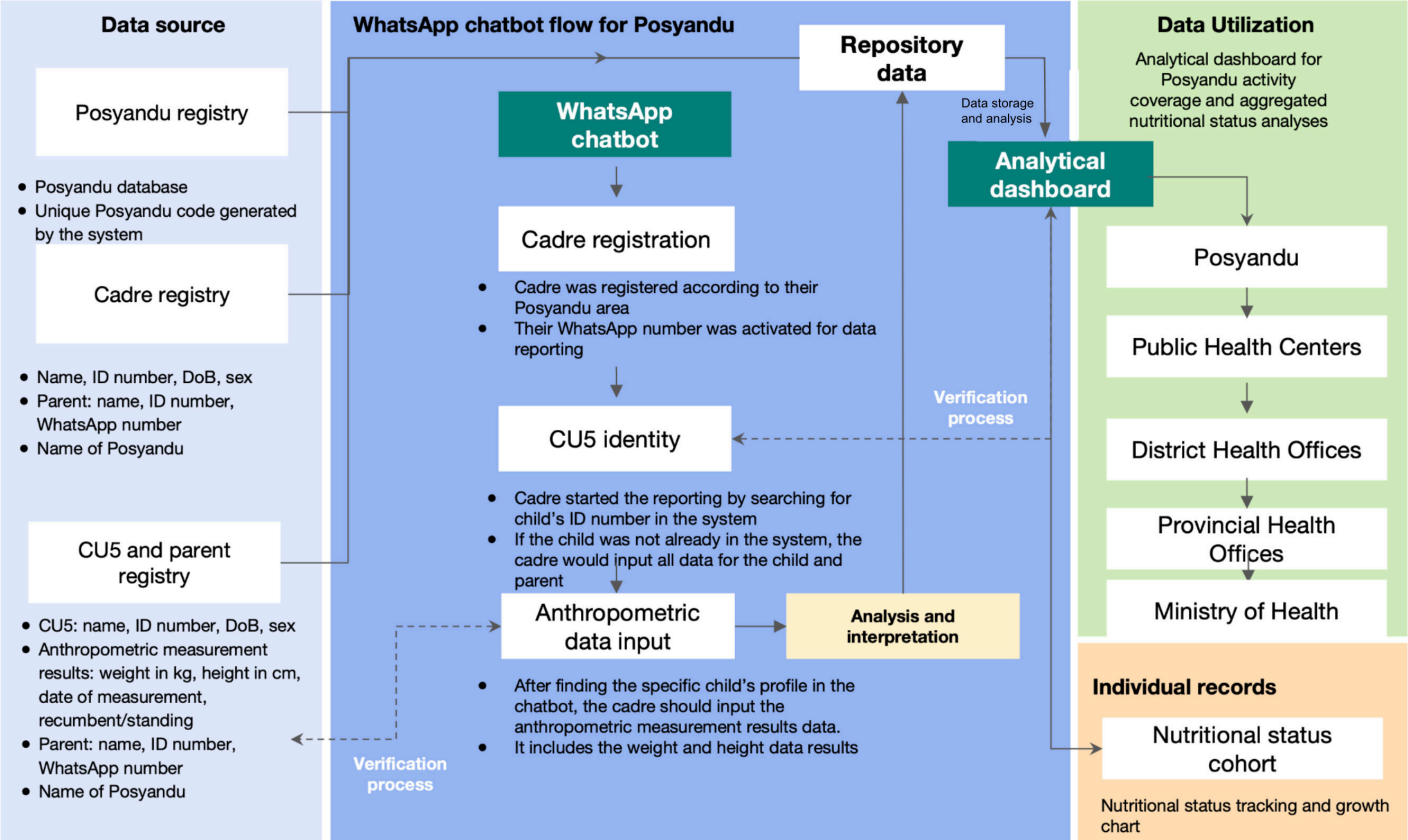
Input, process, and output flow of the WhatsApp chatbot system for nutrition surveillance in Posyandu. CU5: children under 5 years; DoB: date of birth.

The process of recording the nutritional data using the WhatsApp chatbot involved several stages (Figure 1).

#### Gathering the Basic Data of CU5 and Parents or Caregivers

Basic data were gathered about the CU5 and their parents, including the child’s full name, national identification number (Nomor Identitas Kependudukan; NIK), date of birth, gender, parent’s name and ID number, and lastly the Posyandu ID. For the parent’s data, it was only asked that either the father or mother’s information was entered; in some cases, this information would be that of a grandmother or another caregiver. This manual dataset was then imported into the system.

#### Cadre Registration

The account registration was done to enable the cadres to record the data in the WhatsApp chatbot. The registration required the cadres’ data including their full name, ID number, date of birth, the name of Posyandu they work in, and an active WhatsApp number. This data already existed at Puskesmas, and it was used to validate the input data. The registered cadres’ profiles could be monitored through the operational dashboard, which also served as the user management feature for the WhatsApp chatbot. Only DTO-Pusdatin Ministry of Health and Puskesmas Ciderum Teams had the authority to register and edit the cadres’ accounts.

#### Data Input and Validation

**Figure 2.**
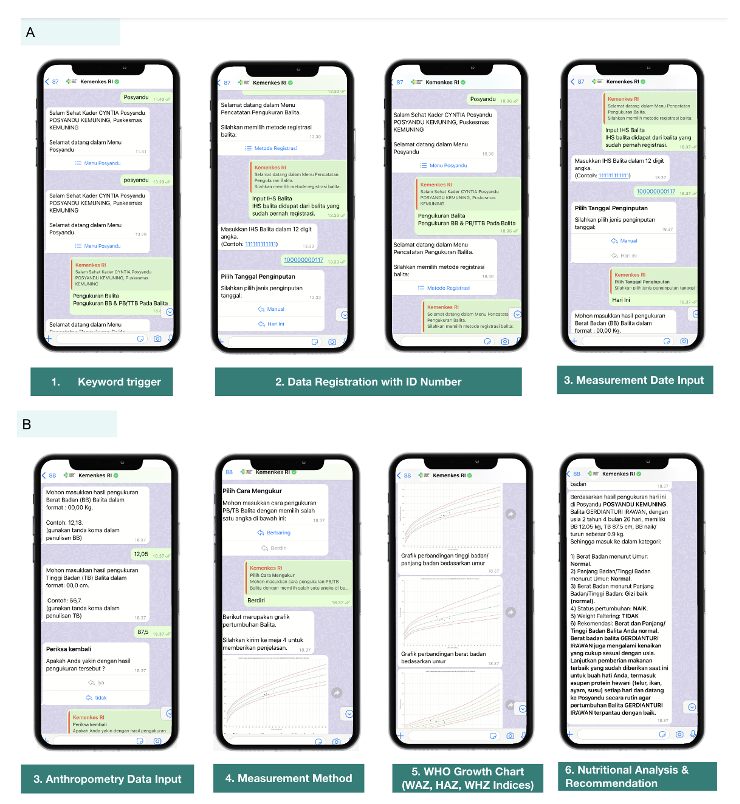
The WhatsApp chatbot interface including (A) starting the flow and child identity data input and (B) anthropometry data input, WHO growth chart, and results analysis. HAZ: height-for-age z-score; WAZ: weight-for-age z-score; WHO: World Health Organization; WHZ: weight-for-height z-score.

After the cadres were registered and their WhatsApp number was activated, the cadres could start to report data by sending “posyandu” as the trigger keyword to activate the data reporting flow of the chatbot (Figure 2). Once the flow started, the variables that should be filled in by the cadres were separated into 3 main sections: child’s data, parent/caregiver’s data, and measurement data. For each variable, automatic validations were set in order to prevent human error in data recording.

For the first section, the child’s name, ID number, date of birth, and sex were required. For the parent’s section, their name, their ID number, and an active WhatsApp number were required. Both the child’s and parent’s ID number were nonmandatory, as some of them may not have had an ID number. Lastly, the measurement section required the date of measurement, weight result (kg, 2 decimals), height result (cm, 1 decimal), and method of measuring height (recumbent or standing).

#### Individual Nutritional Status Analysis

After the cadres completed inputting the requested data, the chatbot system automatically sent the results of 3 nutritional status indices, which included the weight-for-age z-score (WAZ), height-for-age z-score (HAZ), and weight-for-age height z-score (WHZ) (Textbox 1).

Each recorded CU5 was provided with an evaluation of their nutritional status using 3 nutritional indices, accompanied by a growth chart for each index. These growth charts are similar to the World Health Organization (WHO) growth chart, making it easy for the cadres to quickly determine the child’s nutritional status by plotting the growth chart.

Textbox 1. **Nutritional indices and the classifications for each index according to the Ministry of Health Decree Number 2/2020 about Children Anthropometry Standard.**

**1. Weight-for-age z-score (WAZ)**

- Severely underweight (less than –3 SD)
- Underweight (–3 SD to less than –2 SD)
- Normal (–2 SD to +1 SD)
- Risk of overweight (greater than +1 SD)

**2. Height-for-age z-score (HAZ)**

- Severely stunted (less than −3 SD)
- Stunted (–3 SD to less than –2 SD)
- Normal (–2 SD to +3 SD)
- Tall (greater than +3 SD)

**3. Weight-for-height z-score (WHZ)**

- Severely wasted (less than –3 SD)
- Wasted (3 SD to less than –2 SD)
- Normal (–2 SD to +1 SD)
- Overweight (greater than +2 SD to +3 SD)
- Obese (greater than +3 SD)

#### Analytics Dashboard

**Figure 3.**
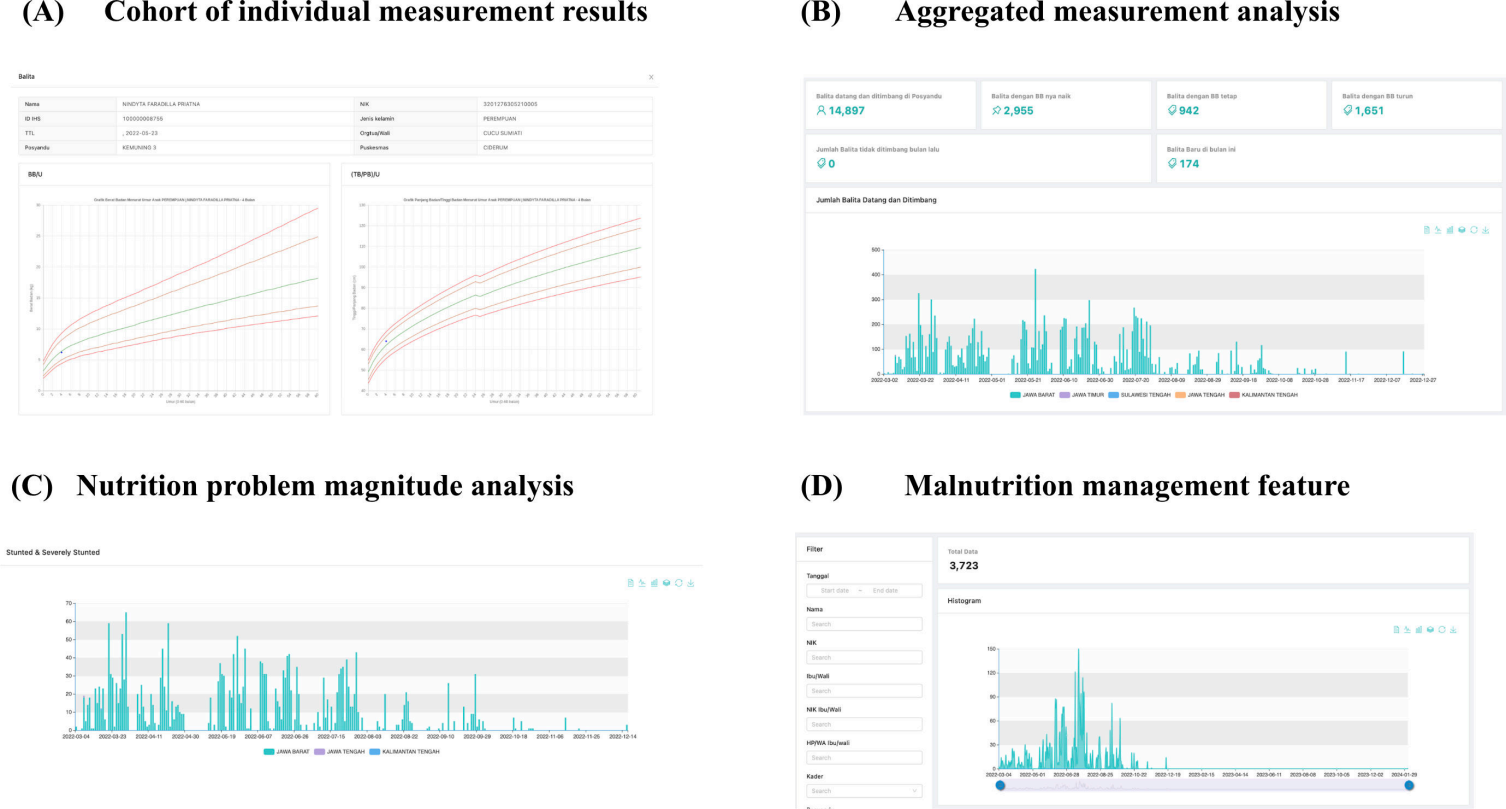
Analytical dashboard features.

In order to provide nutritional status interpretations, the reporting system offered individual recording outcomes and aggregate analyses through a dashboard (Figure 3). The analysis dashboard was divided into four sections, including (1) cohort of individual measurement results, (2) aggregated measurement analysis, (3) analysis of nutritional problem magnitudes, and (4) malnutrition management feature (Figure 3).

#### Excel Download for Cadres

For cadres who completed inputting weighing results at Posyandu, the chatbot flow offered the option to download the recorded data in Excel format. Selecting “YES DOWNLOAD” prompted the system to send an Excel file containing weighing results not only from one cadre but from all cadres who input data for all CU5 attending the same Posyandu within the same time frame. This excel data served as a simple way to check the number of recorded CU5 in the system, especially for the Puskesmas, which mostly required timely reports of recent Posyandu activity.

### System Architecture

**Figure 4.**
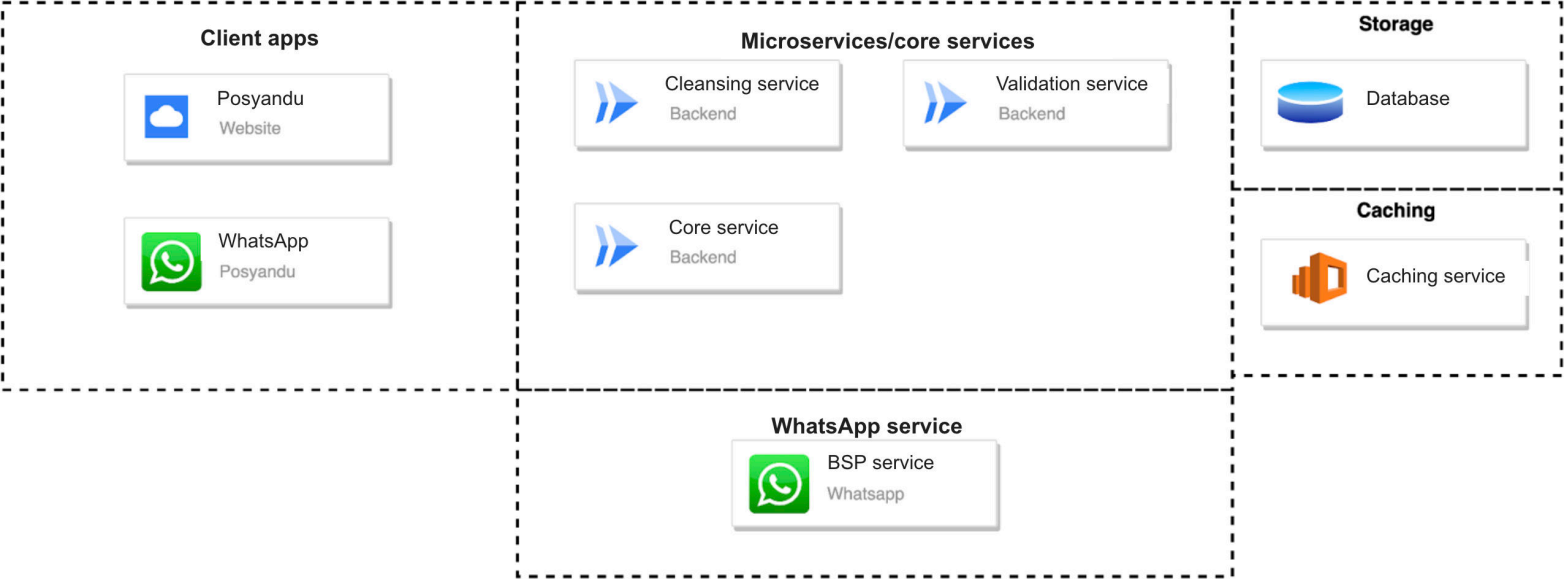
WhatsApp chatbot system architecture. BSP: business service provider.

Client applications, microservices, and the WhatsApp service, storage, and caching are the 5 components that make up the architecture used for this chatbot development (Figure 4). A website and a WhatsApp client (chatbot) are the components that make up the client apps. For the purpose of facilitating the submission of data from the WhatsApp chatbot, the website functions as a front-facing interface for cadres and system administrators. Additionally, it is able to process and change the master data that is going to be used by the WhatsApp chatbot, such as Posyandu data. The information that is stored in each of these front-facing interfaces is stored in a MySQL database. As a result of the large amount of data that is processed, an additional caching service was designed in order to speed up the look up and processing of data within the database.

The microservices model allows for communication between the storage and the front-facing interface. The front-facing client and the database are connected through a number of microservices, which act as the gateway for the exchange of data between the two. In addition, these services incorporate logics and rules that are in accordance with the standards set forth by the World Health Organization (WHO) in order to process the information that is received from the front-facing interface before saving it in the database. Additionally, WhatsApp possesses its very own microservice, which is responsible for communicating the outcome of the computation and the data from the database to the chatbot before it is displayed to the user.

### Data Analysis

**Figure 5.**
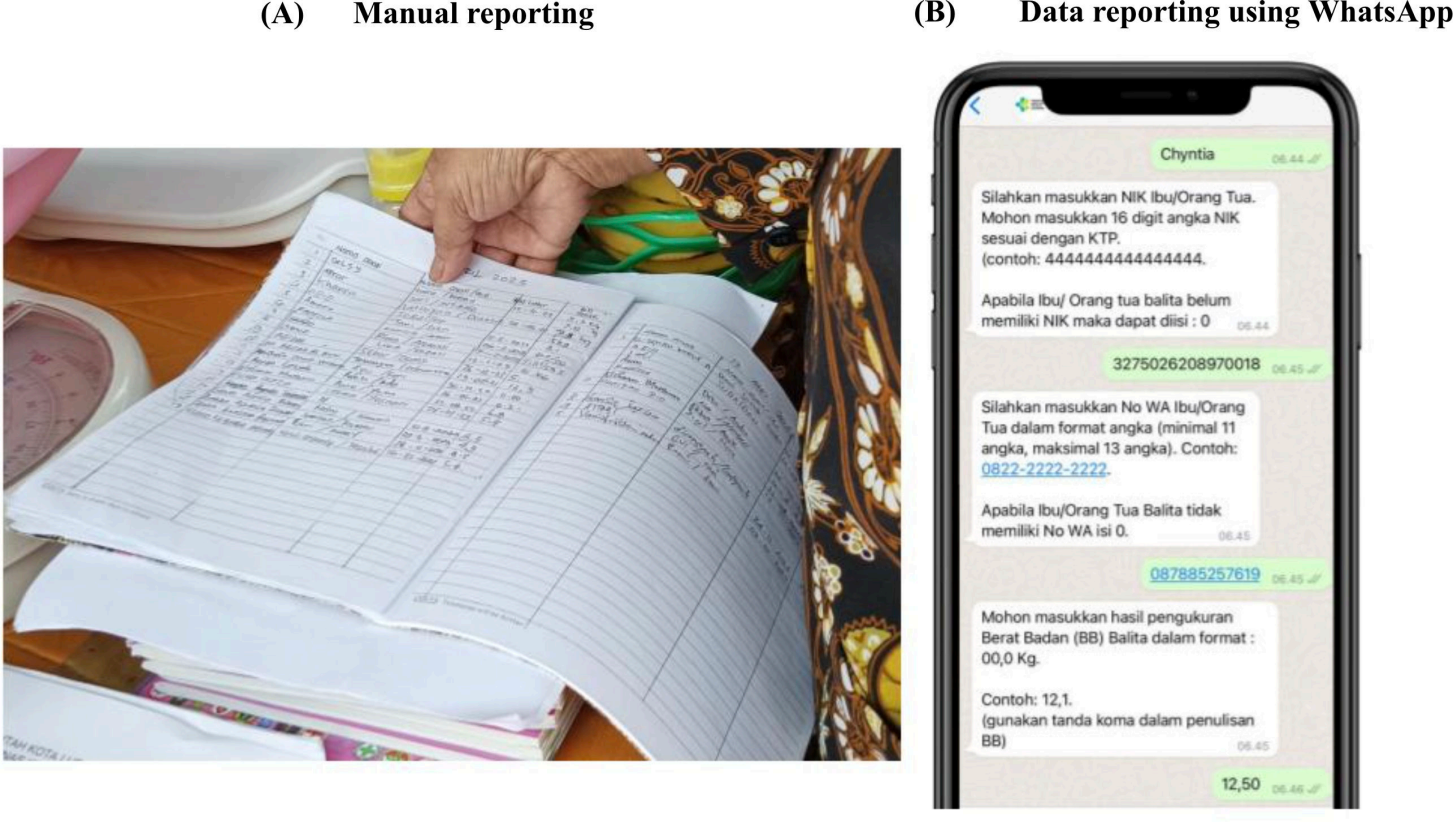
Posyandu reporting before (left) and after (right) using the WhatsApp chatbot.

The cadre entered individual data by entering the child’s 16-digit NIK or Individual Health Status (IHS) number. The cadre was then asked to enter the child’s height and weight measurements, as well as how the measurements were taken (standing or recumbent). After all the data were entered, the system calculated the measurement results to be included in the growth chart (Figure 5): based on body height/length and body weight, based on body height/length and age, and based on body weight and age [34]. After that, recommendations for parents were displayed based on the child’s nutritional status. Data collection was carried out from March 1 to June 30, 2022, at 42 Posyandu in 3 villages in the Puskesmas Ciderum work area in Bogor District, namely Ciderum Village, Ciherang Pondok Village, and Pancawati Village.

Before the recording was carried out by cadres, a training session was carried out for one cadre from each Posyandu, with a total of 42 participants, in February 2022. To make it easier for cadres to carry out training for other Posyandu cadres, a booklet and tutorial video were provided. The system trial phase was carried out for 1 month by visiting the Posyandu in each village and an evaluation was carried out at the end of March. In the following 3 months, assistance was provided at 42 Posyandu with 2 evaluation processes through a WhatsApp coordination group with cadres. The Puskesmas officers monitored the input results through the analysis dashboard.

The results of anthropometric data (weight and height) were automatically processed by the system and presented in a structured dashboard, accessible for Puskesmas, District Health Offices, Provincial Health Offices, and the Ministry of Health at the national level. The data could be queried using a set of predetermined variables. The analyses generated were the frequency and percentage of children being measured based on demographic characteristics (sex, age, domicile address), nutritional status (WAZ, HAZ, WHZ), and spatial analysis on nutritional burden. Trends in service outcome were depicted using time series graphs. The comparative results of Posyandu services at the village level were generated, including analysis by sex and age groups.

### Qualitative Assessment

To collect feedback from users, a series of qualitative assessments were carried out on March 31, May 31, and July 4, 2022, using a roundtable discussion method with a total of 48, 41, and 42 participants in the sessions, respectively [35]. This qualitative assessment was done during monitoring evaluation meetings with health cadres who had been trained and joined the pilot program since February 2022.

The participants of the survey were health cadres from 3 villages (Ciderum, Ciherang Pondok, and Pancawati) in Bogor District. The qualitative assessments started 1 month after the pilot project was implemented, and they were repeated at monthly intervals an additional 2 times. The survey used the Mentimeter online platform, enabling participants to answer and show the cumulative, anonymized results directly. The questionnaire consisted of four sections: (1) system usage, (2) data input, (3) overall user feedback, and (4) cadre’s ability.

### Ethical Considerations

We declare that the data collected for this paper do not require ethical approval as no individual data are presented and informed consent was obtained during the survey implementation. The participants were able to opt out at any point during participation in this study. This exemption is regulated in Indonesia Ministry of Health’s National Guidelines and Standards for Ethical Research and Development in Health year 2017, which states that studies are exempt from the ethical review process due to no or minimal potential risk/harm of the studies, and if the information being used was publicly available, including procedures such as surveys or interviews or observations of public behavior. It further specifies that research that is conducted by a department or agency, with the purpose of studying, evaluating, or assessing the benefits of public programs or services, does not require ethics approval [36]. This study ensured that the entire process adhered to ethical principles, including respecting participants’ autonomy, promoting their well-being, and avoiding any harm. These principles were upheld by obtaining informed consent, maintaining the confidentiality of participants’ information, and minimizing any potential risks. Additionally, no compensation was provided to the participants involved in this study.

## Results

### Overview

This study of a WhatsApp chatbot for nutrition surveillance data recording and reporting was done in 3 villages in Bogor District, West Java Province, Indonesia, specifically Ciherang Pondok, Ciderum, and Pancawati. These 3 villages have different characteristics in terms of sociodemographics, facilities, and level of support by local leaders (Table 1).

**Table 1.** Characteristics of the 3 study area villages.

Characteristics	Ciherang Pondok	Ciderum	Pancawati	Total
Children under 5 years, n (%)	1642 (36)	1352 (30)	1577 (34)	4571
Number of Posyandu	13	14	15	42
Number of cadres	65	56	60	282
Internet infrastructure	Good	Fair	Poor	N/A
Support from village leader	Very good	Neutral	Neutral	N/A

a N/A: not applicable.

### Results of CU5 Posyandu Weight and Height Measurement Services

During the study, cadres successfully adapted to the new WhatsApp chatbot for reporting CU5 height and weight measurements, transitioning from manual to digital recording. Implementing the WhatsApp chatbot for data reporting presented a novel experience for the cadres.

**Figure 6.**
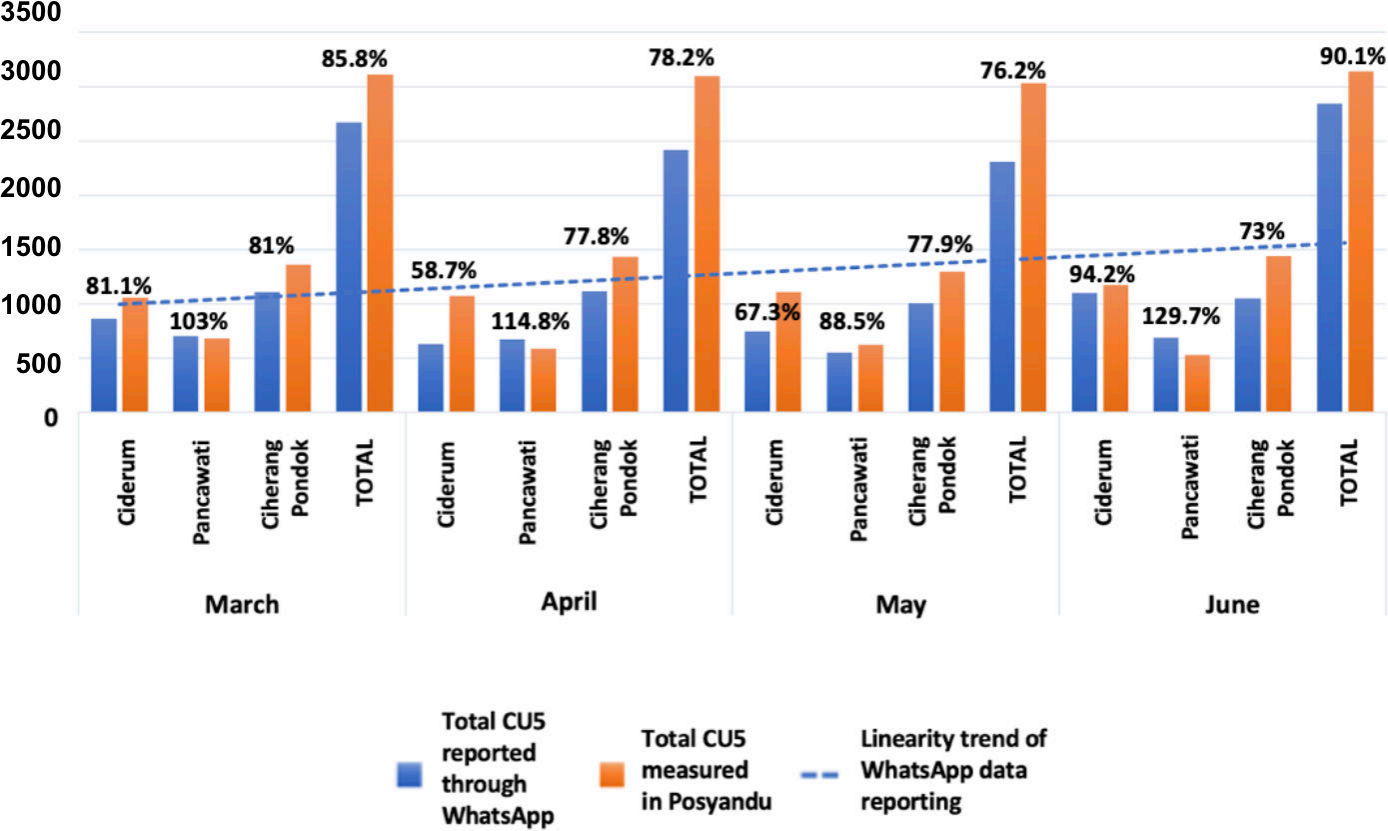
The comparison of total CU5 being measured and those reported through WhatsApp, and the linearity trend of total WhatsApp data reporting month by month. CU5: children under 5 years.

Generally, it was found that the total number of CU5 measurements input into the chatbot was lower than the total number of children weighed at the Posyandu (Figure 6). The highest proportion of measurements input was in June (n=3144, 90.1%), followed by March (n=3113, 85.8%), April (n=3095, 78.2%), and May (n=3032, 76.2%). In April and May, fewer results were entered due to Ramadan and Eid al-Fitr affecting how well cadres could input data. Toward the study’s end, a reminder was sent to cadres by DTO-Pusdatin Ministry of Health and village leaders about data entry.

In terms of each village’s performance of inputting data, Pancawati had the highest number, followed by Ciherang Pondok and Ciderum. However, the data show that Pancawati had more children entered into the system than the number of children actually weighed. In March, April, and June, the percentage of CU5 weighed exceeded 100%, possibly because the chatbot recorded CU5 not just from the monthly weighing but also from those weighed outside the Posyandu area. Additionally, some data might have been entered twice, thus increasing the total number of records, but not affecting individual recordings.

**Figure 7.**
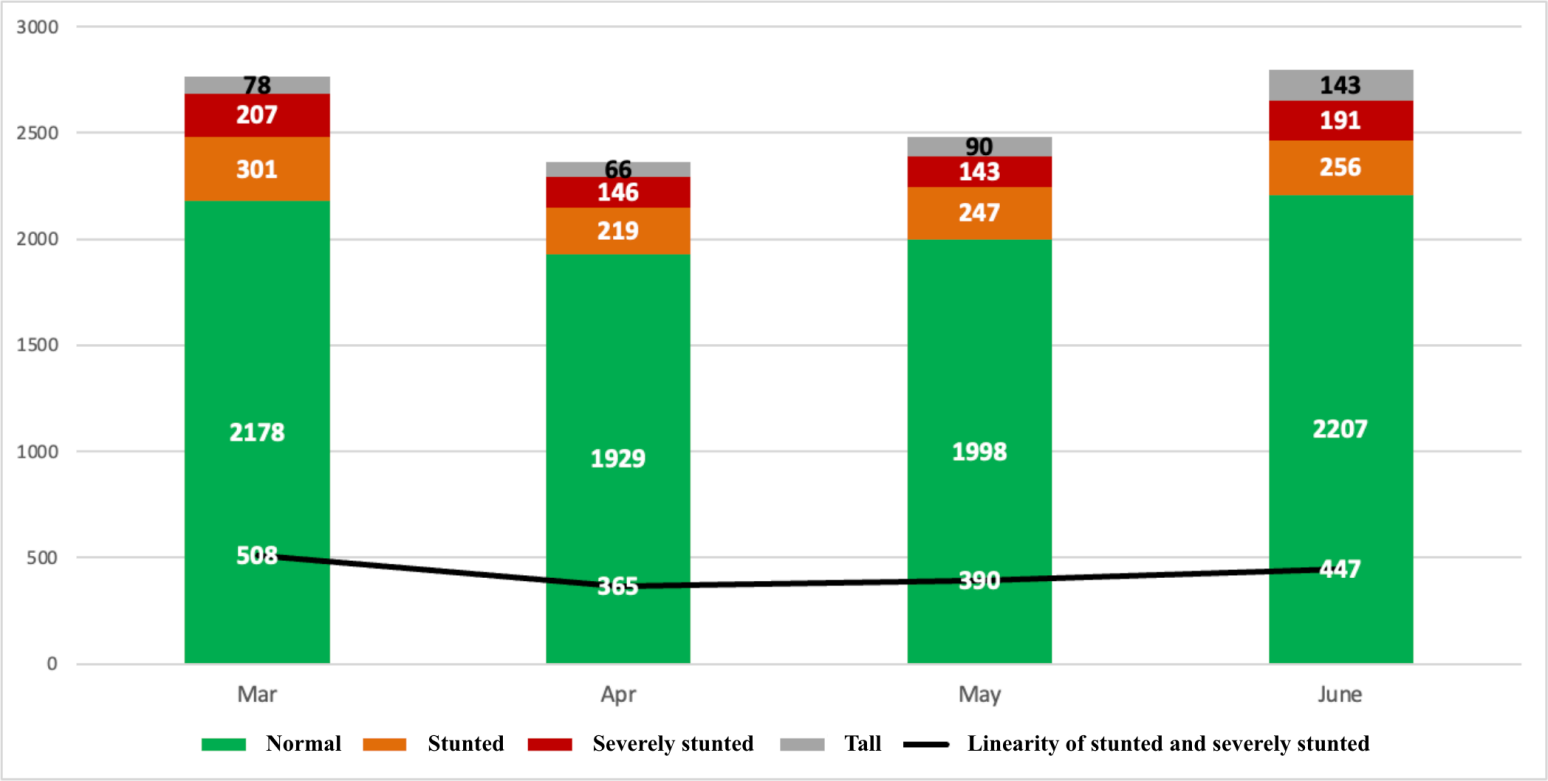
Measurement result data analysis based on the height-per-age z-score.

Further analysis on HAZ was done, categorizing children into severely stunted, stunted, normal, and tall according to the Ministry of Health Decree Number 2/2020 about Children Anthropometry Standard guidelines (Figure 7). In general, the majority of CU5 had a normal nutritional status based on their HAZ compared to other nutritional statuses. Following the “normal” status, the next highest counts were for “stunted,” “severely stunted,” and “tall” statuses. This sequence of HAZ nutritional statuses remained consistent throughout the 4-month observation period.

### User Feedback on WhatsApp Usage for the Posyandu Reporting System From Cadres

The qualitative exploration of the system usage shows that all participants used the system (129/129, 100%). Regarding the data input, half the users (45/90) self-reported that all data had been input into the system, followed by 25.6% (23/90) reporting that three-quarters of the data had been input into the system, and 24.4% (22/90) reporting that half or less of the data had been input into the system.

Most respondents suggested the system was easy to use (118/125, 94.4%) and only 5.6% of respondents (7/125) stated that the system was difficult to use. Regarding the variable in the system, none of the respondents stated that the variable (data required including child and parent identity and weight and height measurements) was difficult to understand. Two-thirds (82/123, 66.7%) of the respondents stated the variable was easy to understand, and 33.3% (41/123) stated the variable was very easy to understand. None of the respondents stated that the flow of data input was difficult to understand. Most respondents (93/123, 75.6%) suggested that the flow of data input was easy to understand and 24.4% (30/123) of respondents said that the flow of data input was very easy to understand. Related to the impact of the usage of the system, 74% (91/123) of respondents suggested that the system was improving Posyandu activities and data reporting quality, while 26% (32/123) suggested that the system only improved data reporting quality.

The other aspects explored in the assessment were about the cadre’s ability to train other cadres about the system. In general, 36.5% (46/126) of respondents stated that they were fairly competent at using the system, and 63.5% (80/126) stated that they were proficient at using the system. Regarding the ability to train the other cadres, 0.8% (1/124) of respondents stated that they were uncertain about their ability to train the other cadres, while 28.2% (35/124) stated that they were capable of training other cadres with preparation. The other 71% (88/124) stated that they were very capable at giving training to the other cadres (Table 2).

**Table 2.** Qualitative exploration about the WhatsApp implementation (N=129).

Characteristics		Values, n (%)	
**System usage by cadres (n=129)**			
	Have used the system	129 (100)	
	Never	0 (0)	
**Total data input (n=90)**			
	One-third of the data	12 (13.3)	
	Half of the data	10 (11.1)	
	Three-quarters of the data	23 (25.6)	
	All data	45 (50)	
**Overall user feedback on system**			
***Ease of use (n=125)***			
	Very difficult to use	0 (0)	
	Difficult to use	7 (5.6)	
	Neutral	0 (0)	
	Easy to use	83 (66.4)	
	Very easy to use	35 (28)	
***Variable (n=123)***			
	Very difficult to understand	0 (0)	
	Difficult to understand	0 (0)	
	Neutral	0 (0)	
	Easy to understand	82 (66.7)	
	Very easy to understand	41 (33.3)	
***Flow of data input (n=123)***			
	Very difficult to understand	0 (0)	
	Difficult to understand	0 (0)	
	Neutral	0 (0)	
	Easy to understand	93 (75.6)	
	Very easy to understand	30 (24.4)	
***Impact of system usage (n=123)***			
	Make things more complicated	0 (0)	
	Neutral	0 (0)	
	Improving data reporting quality	32 (26)	
	Improving Posyandu activities and data reporting quality	91 (74)	
**Cadres’ ability**			
***Ability to use the system (n=126)***			
	Having difficulty	0 (0)	
	Still confused	0 (0)	
	Fairly competent	46 (36.5)	
	Proficient/skillful	80 (63.5)	
***Ability to train the other cadres (n=124)***			
	Unable	0 (0)	
	Uncertain	1 (0.8)	
	Capable with preparation	35 (28.2)	
	Very capable	88 (70)	

### SWOT Analysis

The use of WhatsApp for recording and reporting Posyandu results means that cadres do not need to download new apps, but instead can use a popular existing app. This makes Posyandu recording using WhatsApp easy to use and easy to teach to cadres, thereby speeding up the Posyandu monthly reporting process. This is in line with the evaluation results where, in the first evaluation, cadres still had difficulty inputting Posyandu data; conversely, in the next two evaluations, cadres found it easy to use WhatsApp to report the results of weighing CU5.

Cadres can also input data on toddlers from different Posyandu in the same subdistrict. The Excel data input over the last month remains downloadable and this makes it easier for cadres to report to the village midwife and Puskesmas. Moreover, the digital record helps cadres to plot CU5’s nutritional status in a precise manner, allowing them to produce an accurate plot.

Based on the evaluation results, connection problems are the number one challenge in Posyandu reporting using WhatsApp; Table 3 presents a SWOT (strengths, weaknesses, opportunities, threats) analysis. Limited internet connectivity and the absence of an offline input feature prevented cadres from reporting. Additionally, some cadres could not input data in real time, especially for areas with high target data, with a large volume of work related to inputting data in real time. Finally, there are several features that should be considered for future updates, such as a reminder feature for inputting Posyandu results data, or other features that suit the program’s reporting needs.

**Table 3.** WhatsApp chatbot SWOT^a^ analysis.

Category	Strength	Weakness	Opportunity	Threat
Data input process	Cadres can input data for children from different Posyandu within the same subdistrict (Puskesmas).	It depends on the signal; offline data input is not yet possible.	In cases where the number of target children is not high, real-time data input by cadres is feasible.	Slow responses from the chatbot can discourage cadres from inputting a large amount of data.
System utilization	No need to download an app, simply save the chatbot WhatsApp number.	Limitation on providing various features for user through chatbot flow mechanism	Additional features can be introduced based on the reporting program’s needs.	If incorrect information—such as birth date, gender, and weighing results—is input, it can lead to inaccurate interpretation of nutritional status.
Sustainability	Easy to be taught to cadres with low digital literacy	N/A^b^	The sustainability of Posyandu nutritional surveillance data reporting depends on cross-sectoral collaborations from related ministries and stakeholders to support the Puskesmas and Posyandu in implementing the Maternal and Child Health program and recording and reporting data while ensuring data quality. Stakeholders include the National Population and Family Planning Board, the Ministry of Public Works, and the Ministry of Villages, Development of Disadvantaged Regions, and Transmigration.	Depending on server capacity, scalability considerations are essential for national implementation.
Data quality	Validation and verification during data input to minimize errors	Regular data monitoring and evaluation are necessary to support data reporting.	After completing data entry, there is an Excel file containing the weighing results for the last month, facilitating reporting to the village midwife and Puskesmas.	Cadres often input child data with different national identification numbers and Individual Health Status numbers, leading to potential data duplication for a single child in the database.
Significance of innovation	Data security is maintained as cadres can only input data and cannot edit it.	N/A	The interpretation of nutritional status provided at the end of each recording helps cadres to plot nutritional status accurately.	The performance of the chatbot is influenced by the specifications of the cadre’s mobile phone.

a SWOT: strengths, weaknesses, opportunities, threats.

b N/A: not applicable.

Both support from local regional leaders to encourage cadres to input data and regular monitoring and evaluation of reporting are necessary to encourage cadres to input measurement data. If the recording is complete, cadres can report individual weighing results that have not been reported yet.

Cadres’ ability to carry out anthropometric measurements in the correct way and anthropometric tools that are not available or have not been calibrated were some of the challenges in this trial. Not all cadres have devices with high specifications, which slows down the chatbot’s response. This makes cadres unwilling to input large amounts of data. Double data also often occurs because there are discrepancies when inputting the NIK and IHS numbers. If a cadre enters the wrong gender or date of birth, there will be an error in the nutritional interpretation such that the information obtained will be incorrect. In addition, regarding server capacity, it is necessary to consider how such a program could scale up and be implemented nationally.

## Discussion

### Principal Findings

WhatsApp has been used for various health-related purposes [28-34]. This paper demonstrates the feasibility of WhatsApp as a tool for data input and data recording in childhood health programs in Indonesia, where it can be used for inputting data, real-time monitoring of nutritional status, generating plotted growth charts, and automatically generating reports on the activities and performance of the cadres in Posyandu.

Current WhatsApp utilization includes aiding in the preparation for professional medical licensing examinations and facilitating online-mediated learning [37,38]. It serves as a data collection tool for health questionnaires related to conditions like Williams-Beuren syndrome and acts as a survey tool for patients with COVID-19 and cancer [39,40]. In biomedical and health care settings, it is used to support processes like neurosurgery assessments, to monitor the application of braces for clubfoot, in laboratory management systems, and to facilitate patient-related communication [41-44]. Additionally, it plays a role in health programs, including initiatives for smoking cessation, patient follow-up care, online health information services for the prevention of stunting, and various peer-support interventions [45-48]. Despite its demonstrated versatility in many contexts, there is currently limited utilization of WhatsApp as a data input tool.

During the 4 months of this pilot study, there was a significant narrowing of the gap between the number of CU5 receiving measurements at Posyandu and the number of data points reported by cadres through WhatsApp. Recording Posyandu activities with WhatsApp can be considered user-friendly and practical because the cadres are already familiar with the app, as are large parts of the population [49,50]. The frequent use of WhatsApp has made cadres accustomed to its interface, features, and services, making it easier for them to follow recording and reporting instructions.

Any app featuring a well-designed user interface contributes to user comfort when using the app and WhatsApp has been shown to fulfill the memorability and usability aspect of an app [51]. The architectural design of the WhatsApp client is also crafted to deliver a seamless and user-friendly experience [52,53]. From an economic perspective, WhatsApp provides an easily adoptable alternative solution. The majority of cadres are housewives [54,55]. Although employment status does not affect the motivation of cadre contributions, it can pose a challenge because they tend to have limited resources, such as owning a smartphone with limited internet data for digital recording. WhatsApp is a freely downloadable app, so cadres do not need to incur subscription costs to use it [56,57].

During the final stage of the pilot, 15.9% (n=447) of CU5 were reported as having stunted growth. This number was lower than the 2022 estimated prevalence of children with stunted growth obtained through a national survey in Indonesia, which amounted to 21.6% at the national level and 20.2% for West Java Province [7]. The number was also lower than the prevalence of children with stunted growth in Bogor District, which was reported to have reached 24.9% in 2022 [58]. It is unclear if this discrepancy was due to a more accurate reporting methodology, a lack of recording for the more affected children, or other reasons. However, it demonstrates that, even within a pilot application, the use of this tool provides a representative picture.

However, there are several challenges to its nationwide implementation: access to an internet connection, data validity, system capacity, hardware support, and external support. To activate a conversation with the chatbot, an adequate internet connection needs to be established. Despite the increasing adoption of 4G networks and up to 73% internet penetration, disparities in internet access persist in the eastern part of Indonesia [59].

To mitigate challenges related to internet access, it is crucial to support efforts to expand internet quality across all provinces. Efforts that could reduce this gap in the digital divide include but are not limited to: partnering with local business to provide internet in low-resource settings, engaging with multisectoral stakeholders to develop policy frameworks and funding support for targeted areas, as well as encouraging innovation in technology related to internet access [60,61]. Currently, the Indonesian Ministry of Health is exploring the possibility of cooperation with Starlink (a satellite internet provider) to provide internet access in Puskesmas located in remote, frontier, and outermost areas [62]. Aside from that, continuous collaboration with relevant stakeholders such as the Ministry of Communication and Information Technology is needed.

As an alternative, developing offline features would allow for data entry without an internet connection. A previous study showed that offline features in a mobile app could aid data collection in a decentralized registry [63]. Mobile apps with offline functionality such as Research Electronic Data Capture (REDCap) have also been tested for their usefulness in collecting field survey data in areas with low access to the internet [64]. Additionally, offline functionality could also be used for educational purposes to inform mothers in rural areas about their children’s nutrition status [65].

Several studies pointed out the need for attention to the standard operating procedure for anthropometric measurements (especially for body height, which had the highest technical error of measurement), maintenance of instruments, training, and measurement resampling to ensure the accuracy of the data [62,66,67].

To tackle the disparities in anthropometric instruments across the country, the Ministry of Health is striving to equip all Posyandu with anthropometric devices, aiming to distribute 313,737 anthropometric devices to 303,416 Posyandu nationwide by the end of 2024 [68]. Regular calibration must be carried out by an assigned technician to ensure instrument quality; equipment such as infantometers and stadiometers must be calibrated weekly [69]. Another study showed that, to achieve the same accuracy of anthropometric measurement as a medical student, cadres must be trained intensively and receive regular education [70,71]. Capacity building can be carried out as a means to train cadres to conduct anthropometric measurements and digitally report data [72,73].

In order to record individual data, having a NIK number is an integral part of the reporting process. This identification number is able to provide information on location such as province, city/district, and subdistrict, as well as date of birth [74]. Even so, a subpopulation of around 5.3 million people in Indonesia still do not have access to NIK as an identification number [75]. A study by the Ministry of Home Affairs highlights that disadvantaged groups such as individuals living in poverty, indigenous communities, religious or ethnic minorities, remote populations, older individuals, persons with disabilities, refugees, and individuals with nonpermanent housing or high mobility are those having a hard time accessing and obtaining digital identification [76]. Providing legal identification since birth is an area of focus of the Sustainable Development Goals; therefore, to tackle the gap mentioned above, as well as part of a strategy to participate in providing legal identity for all, civil registration data can be linked and integrated with vital statistics such as birth registration data [77,78].

To prevent data duplication, the government shall implement a central database that connects a unique identifier generated by the Ministry of Home Affairs (NIK), Ministry of Health (IHS number), and Immigration Directorate General (passport number) [79-81]. The system shall implement robust data validation mechanisms that could include automated cross-checking against a central database before final submission. This interoperable check of the central database ensures that an individual’s data are recorded through a single, streamlined thread, effectively reducing the risk of duplication and maintaining health data quality [82].

Considering that anthropometric measurement is carried out as a routine activity in Posyandu, it is crucial to ensure that the system is capable of meeting user needs related to inputting data, and that it has the capability to store, file, abstract, and retrieve records seamlessly [83]. Server availability is also one of the crucial components of infrastructure readiness [84]. Another technicality that needs to be considered is the compatibility of the hardware to the software, meaning that to use WhatsApp, potential users need to at least have a device that supports Android OS 5.0 or iOS 12 [85]. Consequently, older devices that were released before 2015 might not be compatible with WhatsApp [85-87].

Taking into consideration that ease of use affects the adoption of apps and/or health systems, developers also need to ensure that the data reporting flow remains simple and the app size is compact [88,89]. Adding features like reminders for data input and providing visual feedback on data consistency could motivate cadres and improve adherence to data entry protocols. Studies have shown that electronic reminders had a great impact on health worker adherence to implementing certain health protocols [90]. Adding monitoring features and feedback loops can also help in identifying cadres who are facing challenges and ensuring targeted support [90].

Aside from the technical support aspect, external support is also pivotal for the implementation of the data input process. Support from the village midwife, Puskesmas, as well as the village leader plays a role in motivating cadres to use the WhatsApp chatbot and input data. It is undeniable that stakeholders such as professional health workers and governmental bodies have a role to play in adequately managing health information systems [91]. Legislative, regulatory, and coordination support are also deemed to be a prerequisite step for optimal and functional health information systems [83]. Having strong cross-sectoral collaborations with relevant ministries and stakeholders, such as the Ministry of Villages, Development of Disadvantaged Regions, and Transmigration; the Ministry of Home Affairs, the National Population and Family Planning Board, and the National Development Planning Agency (Bappenas), will strengthen the broader implementation.

### Strengths and Limitations

The strength of this study lies in its pioneering exploration of digitizing Posyandu records through WhatsApp, representing the first-ever development of such a method with substantial potential for diverse data input approaches, particularly in low- and middle-income countries. However, certain limitations must be acknowledged: (1) data input at Posyandu is dependent on internet access, (2) data validation relies on the standards of measurement tools and the capacity of the cadre, and (3) there exists a potential for record duplication due to discrepancies in the input of NIK and IHS numbers by the cadres. Nevertheless, this study provides a nuanced understanding of the potential application of WhatsApp as a tool for monitoring the nutritional status of CU5 in developing countries such as Indonesia.

### Conclusions

The utilization of WhatsApp holds promise as a tool for recording surveillance data related to the nutritional status of children. This study provides the first evidence of the implementation of such a tool in community-driven health care settings. In the future, there is potential for expanding the application of WhatsApp to encompass broader data recording functions within health programs. However, such an expansion requires concurrent enhancements in features, involving the preparation of human resources, infrastructure at Posyandu, and robust regulatory support. This entails a comprehensive approach that addresses not only the technological aspects but also considers the human, infrastructural, and regulatory dimensions for the effective integration of WhatsApp into health programs, ensuring its optimal and sustainable use.

## Abbreviations

CU5: children under 5 years old
DTO: Digital Transformation Office
HAZ: height-for-age z-score
IHS: Individual Health Status
NIK: national identification number
Posyandu: Pos Pelayanan Terpadu (community-based health service post)
Puskesmas: Pusat Kesehatan Masyarakat
REDCap: Research Electronic Data Capture
WAZ: weight-for-age z-score
WHZ: weight-for-height z-score
